# Development of an Effective Single-Dose PCV2/CSFV Bivalent Subunit Vaccine Against Classical Swine Fever Virus and Porcine Circovirus Type 2

**DOI:** 10.3390/vaccines13070736

**Published:** 2025-07-08

**Authors:** Yu-Chieh Chen, Wen-Bin Chung, Hso-Chi Chaung, Yen-Li Huang, Chi-Chih Chen, Guan-Ming Ke

**Affiliations:** 1 International Degree Program in Animal Vaccine Technology, National Pingtung University of Science and Technology, Pingtung 912301, Taiwan; ycchen@mail.npust.edu.tw; 2 Research Center for Animal Biologics, National Pingtung University of Science and Technology, Pingtung 912301, Taiwan; wbchung@mail.npust.edu.tw (W.-B.C.); hcchaung@mail.npust.edu.tw (H.-C.C.); 3 Department of Veterinary Medicine, National Pingtung University of Science and Technology, Pingtung 912301, Taiwan; 4 Department of Post-Baccalaureate Veterinary Medicine, Asia University, Taichung 413305, Taiwan; ellis7374365@asia.edu.tw; 5 Graduate Institute of Animal Vaccine Technology, College of Veterinary Medicine, National Pingtung University of Science and Technology, Pingtung 912301, Taiwan

**Keywords:** Porcine circovirus type 2, Classical swine fever, single dose, bivalent subunit vaccine

## Abstract

**Background/Objectives**: Porcine Circovirus Type 2 (PCV2) impairs pigs’ immune systems and increases susceptibility to co-infections, including Classical Swine Fever (CSF), a highly contagious disease listed by the World Organisation for Animal Health (WOAH) as notifiable. Therefore, swine operations in CSF-endemic regions are encouraged to immunize piglets with both PCV2 and CSFV vaccinations. Currently, there is no commercially available bivalent vaccine for PCV2/CSFV. **Methods**: In this study, a total of twenty 4-week-old SPF pigs were administered our formulated PCV2/CSFV bivalent subunit vaccine, containing soluble CSFV-E2 (50 µg) and PCV2-ORF2 (100 µg) antigens with a porcine-specific CpG adjuvant. After 4 weeks of vaccination, all pigs were evaluated for efficacy against PCV2 and CSFV. **Results**: Pigs were only immunized once and showed significantly increased neutralizing or ELISA antibody titers against both viruses four weeks post-vaccination. After viral challenges, vaccinated pigs displayed no clinical signs or lesions and had markedly reduced CSFV and PCV2 viral loads in the serum and tissues compared to controls. **Conclusions**: These results demonstrate that a single dose of the PCV2/CSFV bivalent subunit vaccine is safe and effective in young pigs, induces strong antibody responses, and suppresses viral replication, making it a promising tool for swine disease control and cost-effective vaccination strategies.

## 1. Introduction

Porcine Circovirus Type 2 (PCV2) is a pathogen that affects pigs, leading to immuno-suppression and porcine circovirus-associated diseases (PCVADs). Pigs infected with PCV2 become immunocompromised, and PCV2 damages their immune system [1,2]. When PCV2 is in the incubation period in piglets after PCV2 infection, it increases the pigs’ susceptibility to other viruses and leads to co-infection with other diseases [3,4,5,6]. Recent reports indicate that PCV2 infection promotes Classical Swine Fever Virus (CSFV) replication, both in vivo and in vitro [3]. Classical Swine Fever (CSF) serves as a dangerous disease in the swine industry, caused by CSFV. The highly contagious CSFV can result in a decline in pig production, leading to significant financial implications for the pig industry. In a region-specific report, it was discovered that 73.9% of PCV2-positive clinical samples were also positive for CSFV, indicating that co-infection might worsen the symptoms of CSF and lead to more complicated health issues [7]. However, maternal antibodies in pigs gradually decline since the weaning period, causing piglets to be increasingly susceptible to PCV2 infection in the breeding environment [8]. Additionally, PCV2 can persist for extended periods on various surfaces, facilitating continuous viral transmission within pig herds and sustaining infection pressure throughout their growth period [9,10,11,12,13]. Chronic or subclinical PCV2 infections lead to a weakened immune system in the animals and reduce the effectiveness of other swine vaccines, which increases the risk of pigs being infected with other pathogens [3,14,15,16]. These findings emphasize the importance of addressing co-infection in CSFV-endemic areas, and controlling the interference of PCV2 with other vaccines is crucial for the pig production industry, especially in regions that have large pig densities and persistent outbreaks.

To address the interference of PCV2 with other vaccines, it is essential to implement preventive measures and follow suitable vaccination strategies in pig herds to enhance the immune protection of pigs. Previous studies have shown that PCV2 infection may reduce the neutralizing antibody response after vaccination with the CSF live attenuated vaccine, accordingly raising the risk to CSFV infection [16,17,18]. Importantly, some studies have shown that some subunit vaccines for CSFV, Actinobacillus pleuropneumoniae, and PPV1 retain their protective effects even when administered to pigs exposed to or infected with PCV2 in fields [19,20,21]. Based on the above facts, these findings suggest that subunit vaccines may be less affected by PCV2-induced immunomodulation compared to live vaccines [22]. In this situation, the development and evaluation of multivalent subunit vaccines are particularly relevant in regions where PCV2 is prevalent and may interfere with traditional vaccination strategies. Although the data on global vaccine usage are limited, about 35% of all swine vaccines used were a combination of PCV2 and CSFV vaccines before Taiwan officially declared that CSF was eradicated (Statistics of Passed Animal Biological Drugs for Pig Inspection by the Council of Agriculture in Taiwan, 2022). The figure points out a trend of substantial demand for effective immunization strategies against both pathogens in affected epidemic areas.

Since no commercial bivalent vaccine targeting both PCV2 and CSFV is currently available, this study developed a bivalent subunit vaccine comprising CSFV-E2 and PCV2-ORF2 recombinant proteins. Both proteins were produced using a mammalian cell expression system and formulated with a porcine-specific CpG adjuvant and ISA206 commercial adjuvant. Previous studies indicated CpG motifs have been widely recognized as effective immunostimulatory agents capable of enhancing both innate and adaptive immune responses when used in conjunction with protein-based vaccines [23,24,25,26,27]. In this study, a species-specific CpG adjuvant (United States Patent No.: US 10,117,929 B1) designed by one of the co-authors was employed. This CpG adjuvant may be capable of safety and efficacy in combination with protein antigens or subunit vaccines [28]. Therefore, its inclusion in the formulation of this bivalent subunit vaccine is expected to effectively enhance protective immunity in animals.

The objective of this study was to evaluate the immunogenicity and protective efficacy of this PCV2/CSFV bivalent subunit vaccine in pigs following a single immunization, without the need for booster doses. This single-dose vaccine aims to provide effective dual protection against PCV2 and CSFV, thereby simplifying vaccination protocols in pig farms and reducing associated economic costs.

## 2. Materials and Methods

### 2.1. Construction of Recombinant Proteins CSFV-E2 and PCV2-ORF2

The recombinant CSFV-E2 and PCV2-ORF2 proteins were produced using the ExpiCHO™ expression system (Thermo Fisher Scientific, Grand Island, NY, USA). The CSFV-E2 protein was derived from the E2 gene fragment (nucleotide positions 1021st-2294th) of CSFV subgroup 2.1a (AY526726.1), and the PCV2-ORF2 protein from the ORF2 gene (nucleotide positions 1030th–1734th) of PCV2d (MN510433.1). Both recombinant genes, PTP-E2 and PTP-ORF2, were modified for proper expression and synthesized by Genomics BioSci & Tech Co., Ltd. (New Taipei City, Taiwan). The constructs were inserted into the pcDNA™3.4 vector (Life Technologies, Carlsbad, CA, USA) and transfected into ExpiCHO-S™ cells (Cat. No. A29127; Thermo Fisher Scientific, Carlsbad, CA, USA). Stable ExpiCHO™ cell lines were selected for high-yield and soluble protein production. These proteins were extracted and purified using a Ni-NTA column (HisTrap™ HP, Merck KGaA, Freiburg, Germany) according to the manufacturer’s recommendations. The purified recombinant CSFV-E2 and PCV2-ORF2 proteins were confirmed by Western blot using an E2-specific monoclonal antibody (mAb) WH303 (APHA Scientific, Loughborough, UK) and a PCV2 capsid polyclonal antibody (Invitrogen^TM^, Rockford, IL, USA), respectively. The final product of soluble CSFV-E2 and PCV2-ORF2 proteins was frozen in liquid nitrogen and then stored at −80 °C.

### 2.2. Preparation of PCV2/CSFV Bivalent Vaccine

The formulation of the CSFV/PCV2 bivalent subunit vaccine (TAIWAN Patent No.: TW I841146) included 50 µg of CSFV-E2 and 100 µg of PCV2-ORF2 proteins, which were then adjuvanted with 100 µg of porcine-specific CpG adjuvant (United States Patent No.: US 10,117,929 B1) and dissolved in Montanide™ ISA206VG (SEPPIC, Puteaux, France) in a total volume of 2 mL as one dose. This PCV2/CSFV bivalent subunit vaccine was evaluated for the vaccination protocols on piglets.

### 2.3. Animals Experiment Design

**Figure 1:**
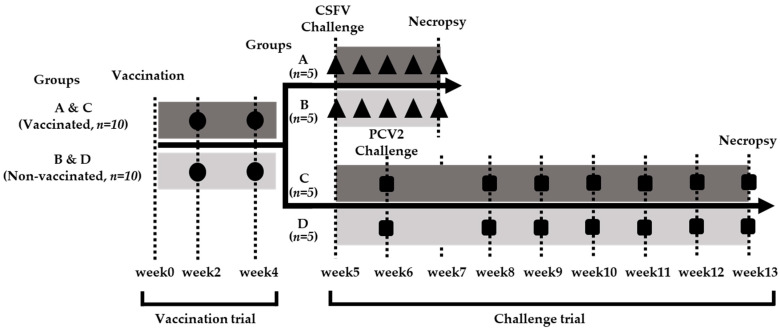
The schematic representation of the animal experimental design. The bleeding times were represented by three symbols: ●, vaccination trial: vaccinated pigs (Groups A and C) and non-vaccinated pigs (Groups C and D) were immunized with the PCV2/CSFV bivalent vaccine or saline, respectively, at week 0 (1st vaccination); ▲, CSFV challenge trial: pigs in Groups A and B were challenged with the CSFV ALD strain (10^5.44^ FAID_50_/total dose per pig) at the beginning of week 5. Blood samples were collected at days 0, 4, 7, 10, and 14 post-challenge, and pigs were sacrificed on day 14 post-challenge (week 7); ■, PCV2 challenge trial: pigs in Groups C and D were challenged with the PCV2d THF0601-7 strain (10^6.90^ TCID_50_/total dose per pig) at the beginning of week 6. Blood samples were collected at weeks 0, 2, 3, 4, 5, 6, and 7 post-challenge, and pigs were sacrificed at week 7 post-challenge (week 13).

The experimental design is illustrated in Figure 1, detailing the vaccination trial, PCV2/CSFV challenge periods, and blood collection time points.

#### 2.3.1. Vaccination Trial

Twenty 4-week-old primary specific pathogen-free (SPF) piglets, reared at the Animal Technology Research Institute (ATRI, Miaoli, Taiwan), were randomly assigned to four groups (*n* = 5 per group) based on body weight and sex. Piglets were delivered via Caesarean section, were colostrum-deprived, and were tested seronegative for anti-CSFV and anti-PCV2 antibodies. Groups A and C received a single 2 mL dose of the PCV2/CSFV bivalent subunit vaccine, while Groups B and D were administered 2 mL of saline (0.9% NaCl). Pigs were monitored for 28 days post-vaccination in a biosafety level II (BSL-2) facility.

#### 2.3.2. Challenge Trials

Four weeks post-vaccination, Groups A and B were challenged with 2 mL of the CSFV ALD strain (10^5.14^ FAID_50_/mL) [29] at the beginning of week 5 following the Animal Drugs Inspection Branch’s guide of Veterinary Research Institute of the Ministry of Agriculture (AHRI, Tamsui, Taiwan). The CSFV challenge was conducted under BSL-3 conditions, with daily clinical monitoring and sample collection regularly under the OIE Reference Laboratory Guidance. At the beginning of week 6 post-vaccination, pigs in Groups C and D were challenged with 2 mL of the PCV2d THF0601-7 strain (10^6^ TCID_50_/mL, Animal Vaccine Labor, National Ilan University, Yilan County, Taiwan). The PCV2d strain was administered intramuscularly once and intranasally for three consecutive days. The challenge trials followed protocols approved by the Institutional Animal Care and Use Committee (IACUC, NPUST, Taiwan, No. 111-010).

#### 2.3.3. Sample Collection and Analysis

Blood samples were collected at defined intervals for serological and viral load analyses. Following the AVMA euthanasia guidelines (2020 edition), routine postmortem tissue sampling was performed. In the CSFV challenge trial, samples included the brain, tonsils, lungs, spleen, hilar (HLN), mesenteric (MLN), inguinal (ILN) lymph nodes, and ileocecal valve (IV). For the PCV2 challenge trial, samples included the lungs, spleen, HLN, MLN, and ILN. Viral loads in the collected tissues were quantified by real-time PCR.

### 2.4. Clinical Examination After CSFV or PCV2 Challenge

After challenge trials with CSFV or PCV2, all pigs were observed daily for clinical signs. Daily clinical inspections for signs of CSF in each animal were monitored from day 1 to day 14 following the CSFV challenge. Clinical scores were recorded and summed daily for 10 parameters by veterinarians for each pig (maximum total score of 30) following the method of Mittelholzer et al. (2000) during the CSFV challenge [30] and using non-parametric statistics to analyze the data. Then, the pigs were recorded and observed daily for PCV2 clinical signs such as body skin, hair, respiratory and digestive symptoms, and activeness according to routine monitoring [31]. Body weight and rectal temperatures were recorded regularly, and fever was defined as a rectal temperature above 40.5 °C according to the criteria demonstrated by Mittelholzer et al. (2000).

### 2.5. Serological Test by ELISA and Virus Neutralization Assay

Blood from all pigs were routinely collected and tested for CSF neutralizing antibodies and ELISA titers of CSF and PCV2 during the animal experiments. Neutralizing antibody titers against the CSFV LPC-TS strain were assessed in all serum samples using a fluorescent antibody virus neutralization test (FAVNT) according to the guidance in Chapter 3.9.3 of the OIE Terrestrial Animal Manual (2022 edition). Commercial ELISA test kits were used to detect antibodies against the CSFV E2-specific antibody (IDEXX Laboratories, Bern, Switzerland) and PCV2 ELISA antibody (BioChek, ER Reeuwijk, The Netherlands) in all serum samples from pig. In the guide book of IDEXX CSFV Ab test kit, results are expressed as blocking percentage, and values greater than or equal to 40% are considered positive. Thus, results were expressed as the S/P ratio, and values greater than or equal to 0.5 were considered positive based on the PCV2 Ab test kit.

### 2.6. Viral Detection and Quantification from Sera and Tissue Samples

CSFV RNA was extracted from the serum and tissue samples using the MagNA Pure 24 Total NA Isolation Kit (Roche Molecular Systems, Inc., South Branchburg, NJ, USA). The viral RNA was quantified following a one-step RT-PCR protocol, based on Hoffmann et al. (2005), with minor adjustments, using the QuantiTect™ Probe RT-PCR Kit (Qiagen, Hilden, Germany) and CSFV-specific primer–probe [32] on a Roche LC480 instrument (Roche^®^, Basel, Switzerland). Cycle threshold (Ct) values were determined using the LightCycler^®^ 480 Software v1.5 to assess the viral loads of CSFV. Quantification was conducted using an OIE Reference Laboratory (New Taipei City, Taiwan) protocol, employing an external standard generated from CSFV ALD strain-challenged blood (10^7.9^ TCID_50_/_mL_), with 10-fold serial dilutions (10^6.9^–10^0.8^ TCID_50_/mL) transformed into Log_10_ TCID_50_/mL or TCID_50_/g for analysis.

The PCV2 genome was detected using RT-PCR following a modified method by Chung et al. (2005) [33]. DNA extraction was performed using the PureLink™ Viral RNA/DNA Mini Kit (Invitrogen^TM^, Carlsbad, CA, USA). The viral DNA of PCV2 was quantified with an external standard containing a defined number of DNA copies, using PCV2-ORF1 primers (GenBank Accession: EU126886). The viral load in each sample was assessed by Ct values, calculated with QuantStudio™ Design & Analysis Software v1.5.1. Quantification was based on 10-fold serial dilutions of the external standard (10^11^−10^−1^ copies/mL), transformed into Log_10_ copies/mL or copies/g for analysis.

### 2.7. Statistical Analysis

Data were expressed as the mean ± SEM of three independent experiments. Clinical scores were compared between groups using the Mann–Whitney U test. Based on methods of Hoffmann et al. (2005) and Chung et al. (2005), the data of CSFV and PCV2 viral loads were quantified using an external standard with a defined number of RNA or DNA copies of a plasmid and transformed as Log_10_ for analysis [32,33]. Comparisons and graphs were analyzed with SAS deployment wizard v9.4 (SAS institute Inc., Cary, NC, USA) and GraphPad Prism 5 (GraphPad Software, Boston, MA, USA). Differences were considered statistically significant for a *p* value less than 0.05.

## 3. Results

### 3.1. ExpiCHO^TM^ Stable Cell Line Expressed Recombinant CSFV-E2 and PCV2-ORF2 Proteins

**Figure 2:**
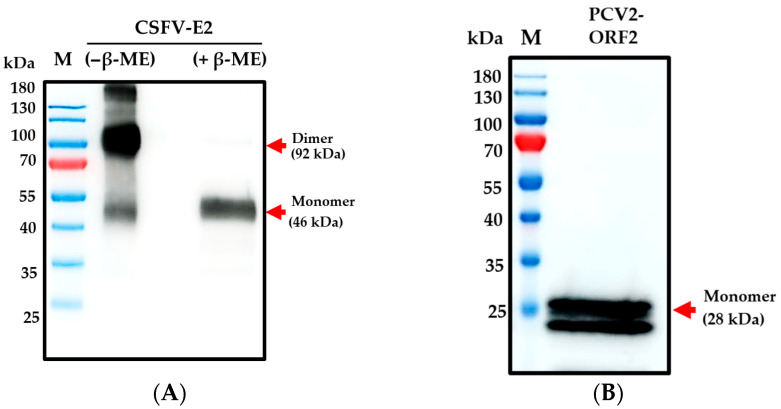
The expression of CSFV-E2 and PCV2-ORF2 proteins from ExpiCHO^TM^ stable cell line. CSFV-E2 (**A**) and PCV2-ORF2 (**B**) proteins were expressed by Western blot. CSFV-E2 protein reduced with or without reagent β-ME (Lane 1 and Lane 2, respectively). Monoclonal antibody (mAb) WH303 and PCV2 capsid polyclonal antibody were used for the Western blot, respectively. Lane M indicates protein markers.

In this study, we have developed a platform and successfully produced soluble CSFV-E2 and PCV2-ORF2 proteins that stably and continuously express the secreted form from the ExpiCHO^TM^ stable cell line. The molecular weight of the mainly expressed native purified recombinant CSFV-E2 protein was about 92 kDa. The monomer under reducing conditions using β-mercaptoethanol (β-ME) appeared to have a molecular weight of 46 kDa (Figure 2A). Further, the monomer of recombinant PCV2-ORF2 protein appeared to have a molecular weight of 28 kDa (Figure 2B). The final product of soluble CSFV-E2 and PCV2-ORF2 proteins was formulated for the PCV2/CSFV bivalent subunit vaccine in this study.

### 3.2. Evaluation of Vaccine-Induced Safety and Immune Response After Vaccination

All examined pigs showed negative results for CSFV E2-specific antibodies and PCV2-ORF2 antibodies before vaccination. The average body temperature (°C) and body weight gains of vaccinated pigs were not significantly different from those of non-vaccinated pigs after vaccination with the PCV2/CSFV bivalent vaccine or saline, respectively.

**Figure 3:**
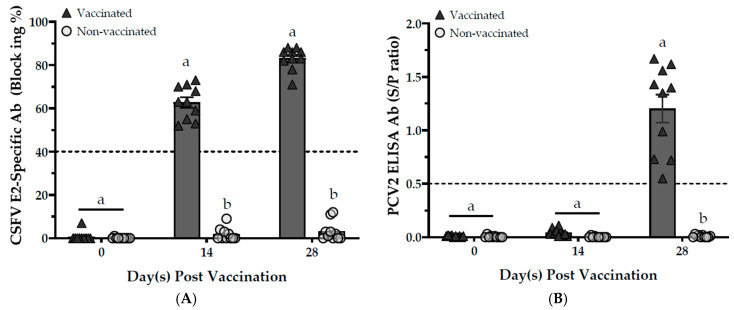
The effect of bivalent vaccine after vaccination in response to CSV or PCV2 sera antibody titers. Sera samples from all pigs were collected and evaluated for CSFV E2-specific Ab (**A**) and PCV2 ELISA Ab (**B**) responses, respectively. Results are values ≥ 40% and ≥ 0.5 that are considered positive by the IDEXX CSFV Ab test (blocking%) and BioChek PCV2 Ab test (S/P ratio), respectively. Data with different letters (^a,b^) indicate that the groups are significantly (*p* < 0.05) different from each other at the same time point.

Four weeks post-vaccination, the sera of vaccinated pigs (Groups A and C) showed an average CSFV E2-specific antibody level of 83.1 ± 1.7 blocking% (Figure 3A) and a PCV2 ELISA antibody level of 1.2 ± 0.1 S/P ratio (Figure 3B). In contrast, the sera of non-vaccinated pigs (Groups B and D) tested negative for both specific antibodies. The data indicate that all pigs vaccinated with the PCV2/CSFV bivalent subunit vaccine produced CSFV-specific and PCV2 antibodies four weeks post-vaccination, resulting in a positive rate of 100% (10/10) after a single vaccination without requiring a booster.

### 3.3. Clinical Presentation Post-Challenge

All pigs were in good health and were divided into groups for the challenge test. In the 14-day CSFV challenge trial, the mean weight gain of pigs in Group B (non-vaccinated) was significantly lower than that of pigs in Group A (vaccinated). The weight of non-vaccinated pigs prior to sacrifice was below the mean weight before the challenge. Similarly, the average weight gain of the pigs in Group D (non-vaccinated) from the PCV2 challenge to before sacrifice was slightly lower than that of the pigs in Group C (vaccinated), although there was no significant difference (Table S1).

**Figure 4:**
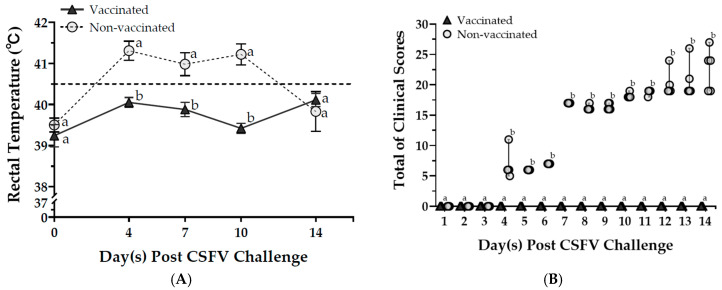
The evaluation of the clinical symptoms of pigs before (day 0) and after the CSFV challenge. Rectal temperatures (**A**) and clinical scores (**B**) were determined in pigs. All pigs were challenged with CSFV (10^5.44^ FAID_50_/total dose per pig) four weeks after vaccination and sacrificed on day 14 post-challenge. Data were expressed as mean ± SEM of rectal temperatures. In addition, the non-parametric Mann–Whitney U test was used to analyze clinical scores, and data were expressed as the median and 95% confidence intervals. Data with different letters (^a,b^) indicate that the groups are significantly (*p* < 0.05) different from each other at the same time point.

In the clinical observations of the CSFV challenge trial, slight classical symptoms were shown by non-vaccinated pigs on the 4th day post-challenge. On day 7, they demonstrated clear signs of depression, anxiety of cold, and unwillingness to move. Some pigs showed neurological symptoms while keeping high fever levels from the 4th day to the 10th day post-CSFV challenge. The mean body temperature of non-vaccinated pigs exceeded 40.5 °C (Figure 4A). Generally, the health status of the non-vaccinated pigs progressively got worse until euthanasia, resulting in a total clinical score of 22.6 ± 1.6. In contrast, the vaccinated pigs exhibited no clinical signs of CSFV after the challenge, maintaining an overall clinical score of 0 during the 14-day period prior to euthanasia (Figure 4B). In the PCV2d strain challenge, the vaccinated pigs did not exhibit observable clinical symptoms, such as fever, weight loss, or respiratory signs. However, non-vaccinated pigs showed mild clinical signs consistent with subclinical PCV2-associated disease, including slightly roughened hair coats and mild diarrhea. Clinical observations supported these results, showing that a single dose of the bivalent PCV2/CSFV subunit vaccine provided partial protection against CSFV and PCV2.

### 3.4. Serological Responses

**Figure 5:**
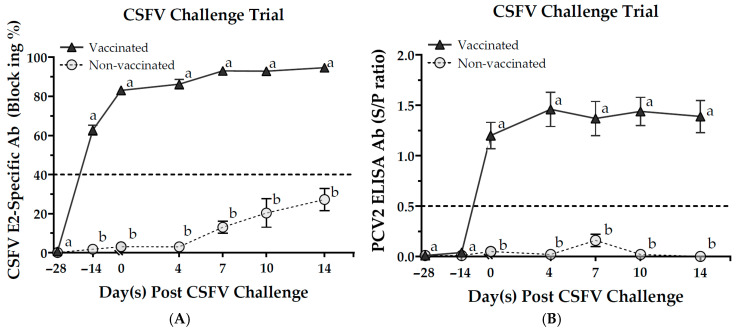

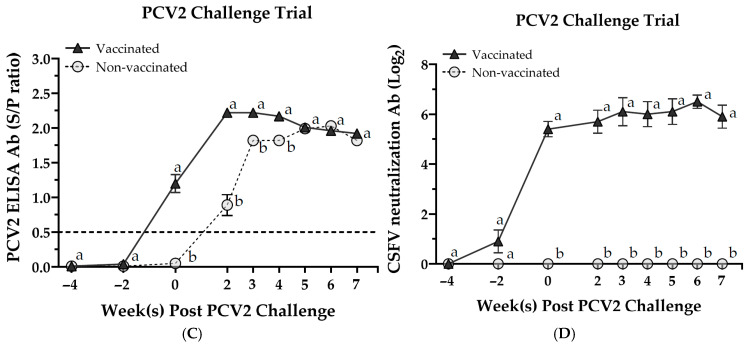
Serological responses in pigs challenged with CSFV or PCV2. Vaccinated (Groups A and C) and non-vaccinated (Groups B and D) pigs received a CSF/PCV bivalent vaccine or saline on day −28 and were challenged on day 0. Sera were analyzed for CSFV E2-specific (**A**), PCV2 ELISA (**B**,**C**), and CSFV-neutralizing (**D**) antibodies. Results are values ≥ 40% and ≥ 0.5 that are considered positive by the IDEXX CSFV Ab test (blocking%) and BioChek PCV2 Ab test (S/P ratio), respectively. Data are presented as mean ± SEM. Different letters (^a,b^) indicate significant differences (*p* < 0.05) at the same time point.

After the CSFV challenge, the levels of CSFV E2-specific antibodies and PCV2 ELISA antibodies in the vaccinated piglets (Group A) remain elevated and showed continued increases, significantly exceeding those observed in the non-vaccinated pigs (Group B) (Figure 5A,B). The levels of CSFV E2-specific antibodies and PCV2 ELISA antibodies in the non-vaccinated pigs were negative.

In the PCV2 challenge trial, vaccinated pigs (Group C) showed an increase in the levels of PCV2 ELISA antibodies after the PCV2 challenge. The antibody titers in this group were significantly higher compared with those of non-vaccinated pigs (Group D) from 2 to 6 weeks post-challenge (Figure 5C). Furthermore, the average titer of CSFV-neutralizing antibodies in vaccinated pigs increased to between (Log_2_) 5.7 ± 0.5 and 6.5 ± 0.3, not being impacted by PCV2 infection. The level of neutralizing antibodies against CSFV remained elevated for a duration of up to 7 weeks (Figure 5D).

The results indicated that, in both CSFV and PCV2 challenge trials, pigs vaccinated once with the PCV2/CSFV bivalent vaccine were able to maintain positive antibody levels against the viruses, specifically for CSFV E2-specific antibodies, PCV2 ELISA antibodies, and CSFV-neutralizing antibodies. Additionally, there was no cross-interference observed in serum antibody expression following viral infection.

### 3.5. Incidence and Amount of PCV2 or CSFV Viral Loads in Serum and Tissue

**Figure 6:**
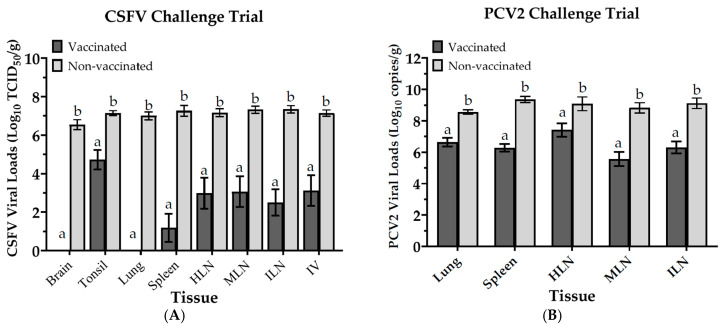
Viral loads in tissue were determined after two challenge trials. Tissues in pigs were collected and examined for viral loads of CSFV (**A**) or PCV2 (**B**) after euthanasia. Data in CSFV challenge trial were expressed as mean ± SEM of log_10_ TCID_50_/g, and in PCV2 trial as mean ± SEM of log_10_ copies/g. Data with different letters (^a,b^) indicate that the groups are significantly (*p* < 0.05) different from each other at the same time point.

In the CSFV challenge trial, the CSFV viral loads in the serum of vaccinated pigs (Group A) were significantly lower than those of non-vaccinated pigs (Group B) at 4, 7, 10, and 14 days after the challenge. In addition, no viral load of CSFV was identified in the serum of the vaccinated pigs from 7 days post-CSFV challenge to the end of the 14-day trial. Among all vaccinated pigs, only one showed a viral titer of Log 2.5 TCID_50_/mL on the 4th day post-challenge, but the days that followed showed no viral residues in the serum (Table 1). The viral loads of CSFV detected in the brain, tonsil, lung, IV and lymph tissue (spleen, HLN, MLN, ILN) samples collected from vaccinated pigs were significantly lower than those in non-vaccinated pigs at necropsy after the CSFV challenge trial (Figure 6A).

Next, the viral loads of PCV2 were determined regularly in the serum of pigs from the second week post-PCV2 challenge to prior to euthanasia. The viral loads of PCV2 in vaccinated pigs (Group C) were significantly lower than those in non-vaccinated pigs (Group D), showing a difference of 1.1 to 6.0 times at 2, 3, 4, and 5 weeks post-challenge. In this trial, the PCV2 viral loads of serum samples from four vaccinated pigs were not detectable after the challenge. However, one of the vaccinated pigs was detected with viral loads of PCV2 in the serum at 4 and 6 weeks post-PCV2 challenge, with higher Ct values of 35 and 37, respectively. And no viral load of PCV2 of serum was present in this pig prior to euthanasia at 7 weeks (Table 2). Furthermore, the viral loads of PCV2 in the lung, spleen, HLN, MLN, and ILN tissue sampling from vaccinated pigs were significantly lower than those from non-vaccinated pigs at autopsy after the PCV2 challenge trial (Figure 6B).

After challenge trials with CSFV or PCV2, pigs administered a single PCV2/CSFV bivalent vaccine showed a significant reduction in CSFV and PCV2 viral loads in both tissues and serum compared to non-vaccinated counterparts, with the complete elimination of viral residues in serum observed. Histopathological analysis showed that vaccinated pigs challenged with CSFV had no lesions in target organs, which further supported these findings. In the PCV2 challenge trial, vaccinated pigs displayed milder histopathological scores (not significant) than non-vaccinated pigs (Figure S1).

## 4. Discussion

Classical Swine Fever Virus (CSFV) and Porcine Circovirus Type 2 (PCV2) are major pathogens of the swine industry that cause substantial economic losses due to decreased productivity, high morbidity, and co-infection complications. Their co-infection poses a significant challenge to herd health, particularly in endemic regions. Several studies have focused on developing PCV2/CSFV bivalent vaccines to enhance herd immunity. However, Huang et al. (2011) reported that PCV2 infection reduces the efficacy of the CSFV live attenuated vaccine, weakening the immune response to CSFV [17]. Additionally, maternal antibodies from sows may interfere with the CSFV live vaccine, leading to insufficient protective immunity in piglets [34,35,36]. To address this issue, Chen et al. (2023) proposed a PCV2/CSFV bivalent subunit vaccine as an alternative to live attenuated vaccines [22]. While previous studies indicate that pigs receiving PCV2-ORF2 subunit vaccines often required a booster immunization three weeks after the primary dose to induce optimal neutralizing antibody responses 3–6 weeks post-immunization [37,38]. Similarly, CSFV E2 subunit vaccines typically require booster doses to achieve over 80 blocking% of CSFV-specific antibodies [39,40,41,42,43,44]. In early experimental studies, some bivalent subunit vaccine formulations produced using Chinese hamster ovary (CHO) cell expression systems or baculovirus expression systems required two doses to enhance antibody responses to PCV2 and CSFV [22,45]. In contrast, our study employed naive, specific-pathogen-free (SPF) pigs and evaluated protective efficacy following a single immunization with a bivalent PCV2/CSFV subunit vaccine. This study demonstrated that the PCV2/CSFV bivalent subunit vaccine elicited a strong antibody response against PCV2 and CSFV-E2 post four weeks, effectively reducing viral nucleic acid levels in serum and tissues, with no detectable virus in the serum and no overt clinical signs. The vaccine provided protection against both the CSFV ALD strain and the PCV2d THF0601-7 strain, supporting its efficacy without the need for booster doses. These findings provide a foundation for future studies that aim to extend the duration of protection and test efficacy under field conditions with maternally derived antibodies. Although further field trials are necessary to assess long-term immunity and achieve applicability, the simplified vaccination protocols observed here indicate possibilities for reduced labor costs and enhanced herd compliance, making it a promising tool for swine disease management.

The development of subunit vaccines relies on various protein expression systems, ranging from prokaryotic to eukaryotic platforms, each with distinct advantages and limitations. Escherichia coli, a widely used low-cost expression system, offers rapid growth and high protein yields but presents challenges such as incorrect protein folding, a lack of glycosylation, and the formation of insoluble inclusion bodies, necessitating solubilization and refolding steps, which increase purification costs compared to eukaryotic cell expression systems [46,47,48]. Many antigens require post-translational modifications (PTMs), such as glycosylation or methylation, which are essential for protein folding, stability, and immunogenicity [49,50,51]. To address these limitations, eukaryotic expression systems, including insect and mammalian cells, are preferred due to their ability to accurately perform PTMs [52,53]. However, achieving high antigen expression levels remains a key challenge in eukaryotic systems. To enhance protein yields, optimized baculovirus–insect cell expression systems have been developed, achieving PCV2 ORF2 protein yields ranging from 56 mg/L to 200 mg/L and CSFV E2 protein expression levels of 30 mg/L to 65 mg/L [54,55,56]. Similarly, mammalian cell lines, including baby hamster kidney (BHK-21), porcine kidney (PK15), human embryonic kidney (HEK293), and Chinese hamster ovary (CHO) cells, have been successfully used to express PCV2 ORF2 and CSFV E2, with yields ranging from 40 mg/L to 150 mg/L [57,58,59,60].

In this study, PCV2-ORF2 and CSFV-E2 recombinant proteins were expressed using an optimized ExpiCHO^TM^ cell expression system to formulate a PCV2/CSFV bivalent subunit vaccine. The CSFV-E2 glycoprotein, in particular, requires eukaryotic expression to ensure proper folding and PTMs, which are critical for immunogenicity [61,62]. Furthermore, Xu et al. (2023) highlighted the dimeric structure of CSFV-E2 as a key factor in eliciting a robust immune response [63]. Interestingly, the CSFV-E2 protein retained this dimeric structure (Figure 2) in this study and the optimized CHO cell expression platform enables the efficient secretion of recombinant proteins into the culture supernatant, facilitating simple purification using Ni affinity chromatography. This system produces correctly folded, functionally active, and highly soluble target proteins, demonstrating its potential for large-scale subunit vaccine production against various infectious diseases.

Subunit vaccines may offer a promising alternative to address the immunosuppressive effects of PCV2 and thus reduce the efficacy of live attenuated vaccines [17,64]. Despite their advantages, the large-scale production of subunit vaccines remains a significant challenge due to the high costs associated with generating immunogenic proteins. In this study, the bivalent PCV2/CSFV subunit vaccine evaluated required only 100 µg of PCV2-ORF2 protein per dose. Notably, this formulation maintained PCV2-specific antibody positivity in pigs for 4–11 weeks post-immunization. Moreover, no significant differences in antibody titers were observed following the PCV2 challenge, indicating stable and effective immune protection (Figure 5C). In comparison, previous studies have shown that immunization was necessary with 200 µg of the baculovirus-expressed PCV2-ORF2 protein to enhance the positive levels of PCV2-specific antibodies [37,65,66]. Furthermore, some studies have demonstrated that, while vaccinated pigs showed elevated serum IgG levels four weeks post-immunization, antibody titers in some individuals declined by approximately 2–5-fold between weeks 8 and 12 in the field trials [65]. Similarly, the PCV2-ORF2 protein expressed in mammalian systems required a 150 µg dose in mice to achieve adequate immunogenicity [60].

For CSFV-E2 subunit vaccines, most immunization strategies require at least 75 µg of E2 protein, with CSFV-E2 antibody positivity occurring 4–5 weeks post-vaccination [43,67]. Li et al. (2020) demonstrated that immunizing 5-week-old piglets with 80 µg of CHO-S-expressed CSFV-E2 protein resulted in CSFV-E2 blocking rates of 65–80% after five weeks [41]. In contrast, the PCV2/CSFV bivalent subunit vaccine in this study contained only 50 µg of CSFV-E2 protein, yet a single immunization induced over 60% E2 antibody positivity within two weeks, with levels rising to 83% blocking at four weeks and 95% blocking thereafter (Figure 5A). The vaccinated pigs had minimal to no viremia, no significant clinical symptoms, and smaller or even no pathological lesions compared to the non-vaccinated group.

These results suggest that this PCV2/CSFV bivalent subunit vaccine with reduced antigens effectively elicits immune responses following a single dose compared to the formulations of previous studies. This bivalent subunit vaccine holds strong potential to maintain protective immunity in future field applications and represents a cost-effective and efficient strategy for controlling PCV2 and CSFV infections in swine populations.

## 5. Conclusions

In summary, pigs immunized with a single dose of the PCV2/CSFV bivalent subunit vaccine developed strong humoral responses, with the evidence of protection against viremia and clinical signs, despite detectable residual viral loads in tissues. Four weeks post-immunization, 100% of vaccinated pigs were positive for CSFV E2-specific antibodies, CSFV-neutralizing antibodies, and PCV2 ELISA antibodies. Furthermore, following CSFV or PCV2 challenge, vaccinated pigs exhibited no clinical signs or external lesions. The viral nucleic acid levels of CSFV and PCV2 in tissues were significantly suppressed, and viremia was effectively prevented. These findings demonstrate that this single-dose PCV2/CSFV bivalent vaccine not only provides effective protection against both pathogens but also offers a cost-efficient solution for swine health management by reducing the need for multiple vaccinations and minimizing antigen usage.

## 6. Patents

This PCV2/CSFV bivalent subunit vaccine has a TAIWAN patent number (TW I841146), and we have applied for its United States patent. These are the patents resulting from the data in this manuscript. Porcine-specific CpG adjuvant has a United States Patent number (US 10,117,929 B1), and it was included in this bivalent subunit vaccine.

## Figures and Tables

**Table 1:** Viral loads in sera of pigs challenged with CSFV.

Group (*n*) *	Vaccinated (5)		Non-Vaccinated (5)	
	Ct	Log_10_ TCID_50_/mL	Ct	Log_10_ TCID_50_/mL
Day(s) Post CSFV Challenge (dpv)				
−28 dpv	(−)	(−) ^a^	(−)	(−) ^a^
−14 dpv	(−)	(−) ^a^	(−)	(−) ^a^
0 dpv	(−)	(−) ^a^	(−)	(−) ^a^
4 dpv	32.0 **^⁘^**	0.5 ± 0.5 ^a^	25.2 ± 0.8	4.6 ± 0.2 ^b^
7 dpv	(−)	(−) ^a^	16.9 ± 0.6	7.2 ± 0.2 ^b^
10 dpv	(−)	(−) ^a^	13.8 ± 0.8	8.1 ± 0.2 ^b^
14 dpv	(−)	(−) ^a^	14.7 ± 0.4	7.8 ± 0.1 ^b^

* Vaccinated pigs (group A) and non-vaccinated pigs (group B) were immunized with PCV2/CSFV bivalent vaccine or saline, respectively, on days −28 (1st vaccination). All pigs were challenged with CSFV (10^5.44^ FAID_50_/total dose per pig) four weeks after vaccination and sacrificed on day 14 post-challenge. Data are presented as Ct values, transformed into mean ± SEM of Log TCID_50_/mL. Data superscript letters (^a,b^) indicate statistically significant differences between groups at the same time point (*p* < 0.05); (−): non-detectable by qPCR. ^⁘^ Only one of the three pigs was detectable, resulting in an incompatible data format; no statistical comparison could be made.

**Table 2:** Viral loads in sera of pigs challenged with PCV2.

Group (*n*) *	Vaccinated (5)		Non-Vaccinated (5)	
	Ct	Log Copies/mL	Ct	Log Copies/mL
Week(s) Post PCV2 Challenge (wpv)				
−4 wpv	N/A	(−) ^a^	N/A	(−) ^a^
−2 wpv	N/A	(−) ^a^	N/A	(−) ^a^
0 wpv	N/A	(−) ^a^	N/A	(−) ^a^
2 wpv	N/A	(−) ^a^	32.8 ± 0.8	5.2 ± 0.2 ^b^
3 wpv	N/A	(−) ^a^	31.7±0.6	6.0 ± 0.2 ^b^
4 wpv	35 **^⁘^**	0.9 ± 0.9 ^a^	31.4 ± 1.3	5.6 ± 0.4 ^b^
5 wpv	N/A	(−) ^a^	35.2 ± 0.8	5.7 ± 0.2 ^b^
6 wpv	37 **^⁘^**	1.0 ± 1.0 ^a^	38.7 ± 0.3	1.8 ± 1.1 ^a^
7 wpv	N/A	(−)	39 **^⁘^**	0.9 ± 0.9 ^a^

* Vaccinated pigs (Group C) and non-vaccinated pigs (Group D) were immunized with the PCV2/CSFV bivalent vaccine or saline, respectively, on days −28 (1st vaccination). All pigs were challenged with PCV2 (10^6.90^ TCID_50_/total dose per pig) four weeks post-vaccination and sacrificed at week 7 post-challenge. Data are presented as Ct values, transformed into mean ± SEM of Log_10_ copies/mL. Data superscript letters (^a,b^) indicate statistically significant differences between groups at the same time point (*p* < 0.05); (−): non-detectable by qPCR. **^⁘^** Only one of the three pigs was detectable, resulting in an incompatible data format; no statistical comparison could be made.

## Data Availability

The raw data supporting the conclusions of this article will be made available by the authors on request.

## Supplementary Materials

The following supporting information can be downloaded at: https://www.mdpi.com/article/10.3390/vaccines13070736/s1, Table S1: Mean body weight gain of pigs after vaccination and challenge with CSFV or PCV2. Figure S1. The histopathological lesions were scoring in pigs after two challenges trails.

## Abbreviations

The following abbreviations are used in this manuscript:

PCV2	Porcine Circovirus Type 2
CSF	Classical Swine Fever
CSFV	Classical Swine Fever Virus
WOAH/OIE	World Organisation for Animal Health
SPF	Specific pathogen-free
BSL	Biosafety level
HLN	Hilar lymph node
MLN	Mesenteric lymph node
ILN	Inguinal lymph node
IV	Ileocecal valve
