# Supplementary material for: Development of an Effective Single-Dose PCV2/CSFV Bivalent Subunit Vaccine Against Classical Swine Fever Virus and Porcine Circovirus Type 2
Source: Vaccines (Basel). 2025 Jul 8;13(7):736. doi: 10.3390/vaccines13070736 (PMC12299614; doi:10.3390/vaccines13070736)
Supplement: Supplementary file 1 [file vaccines-13-00736-s001.zip › Table S1_Mean body weight gain of pigs after vaccination and challenged with CSFV or PCV2..pdf]

Table S1. Mean body weight gain of pigs after vaccination and challenged with CSFV or PCV2.

Animal Trials[*]	Mean body weight gain[**] (kg)	
	Vaccinated (*10*)	Non-vaccinated (*10*)
Vaccination trial	6.9±0.2[a]	7.1±0.2[a]
CSFV challenge trial	9.1±1.0[a]	-1.0±0.5[b]
PCV2 challenge trial	31.2±1.8[a]	26.8±2.3[a]

[*] In vaccination trial, all pigs were immunized with PCV2/CSFV bivalent vaccine or saline respectively on days -28, and challenged with CSFV or PCV2 on day 0. A duration of 14 days and 7 weeks in CSFV and PCV2 challenge trials, respectively. Vaccinated pigs (groups A and C; n=10) and non-vaccinated pigs (groups B and D; n=10) were immunized with CSF/PCV bivalent vaccine or saline, respectively. Pigs were challenged with CSFV (groups A and B) or PCV2 (groups C and D) four weeks after vaccination.

[**] Body weights were determined before or after vaccination and challenge for calculating mean body weight gains. Data were expressed as mean ± SEM.
