# Supplementary figures and images for: Development of an Effective Single-Dose PCV2/CSFV Bivalent Subunit Vaccine Against Classical Swine Fever Virus and Porcine Circovirus Type 2
Source: Vaccines (Basel). 2025 Jul 8;13(7):736. doi: 10.3390/vaccines13070736 (PMC12299614; doi:10.3390/vaccines13070736)

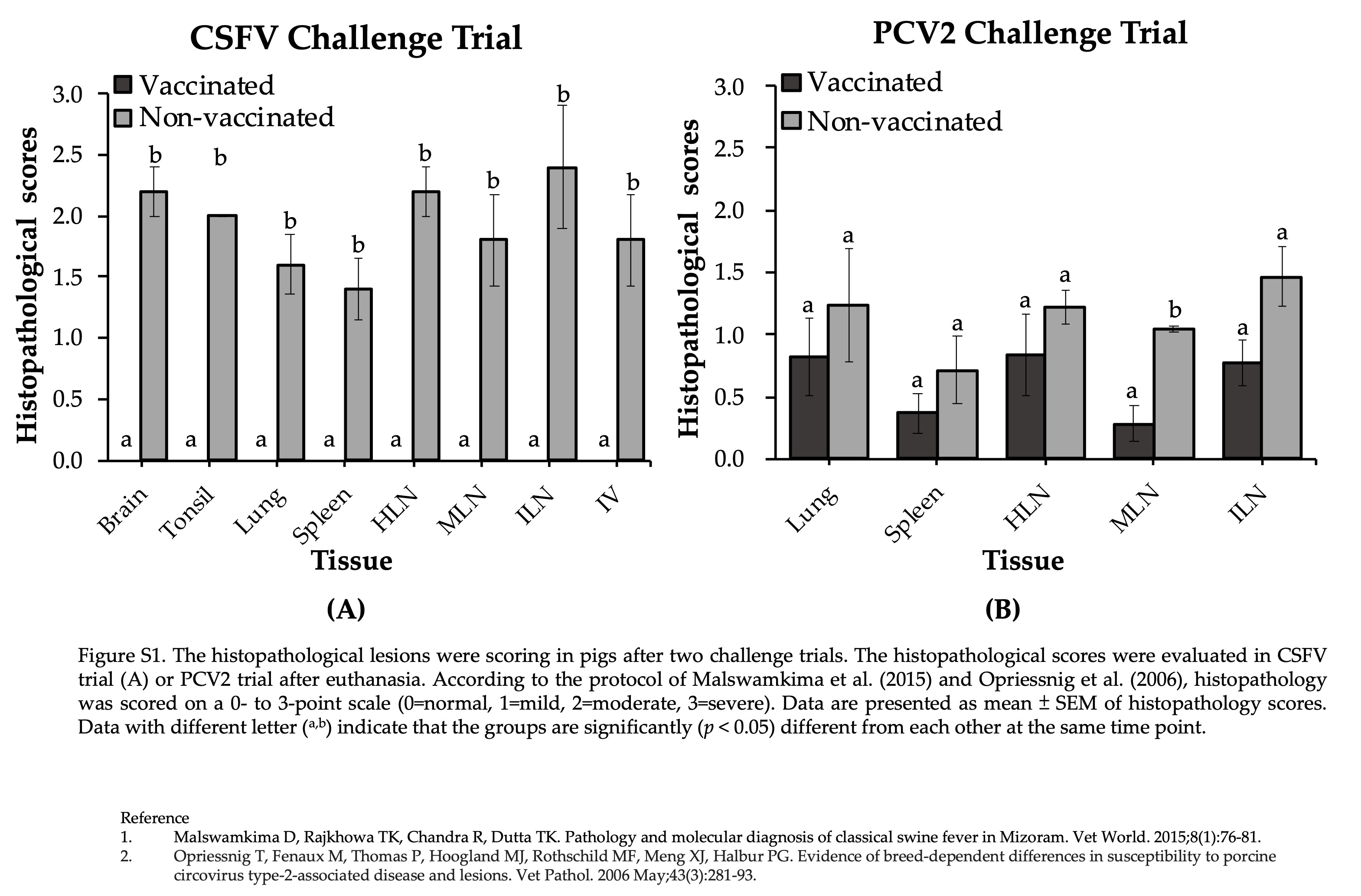

Supplement: Supplementary file 1 [file vaccines-13-00736-s001.zip › Figure S1. The histopathological lesions were scoring in pigs after two challenge trials. .jpg]
